# Efficacy evaluation of a fermented milk containing inactivated biomasses of *Lacticaseibacillus rhamnosus* and *Saccharomyces cerevisiae* to ameliorate the toxic effects of dietary aflatoxins and fumonisins in pigs: a pilot study

**DOI:** 10.1007/s12550-026-00677-7

**Published:** 2026-09-04

**Authors:** Lucas Gabriel Dionisio Freire, Jennifer Rafaela Sá da Silva, Lucas Luiz Assis, Leandra Náira Zambelli Ramalho, Fernando Silva Ramalho, Fabio Enrique Lemos Budiño, Karina Nascimento Pereira, Rogério D’Antonio Pires, Sana Ullah, Sher Ali, Giovana Fumes Ghantous, Roice Eliana Rosim, Carlos Humberto Corassin, Carlos Augusto Fernandes Oliveira

**Affiliations:** 1 Department of Food Engineering, School of Animal Science and Food Engineering, University of São Paulo, Pirassununga, SP Brazil; 2 Department of Pathology and Legal Medicine, School of Medicine at Ribeirão Preto, University of São Paulo, Ribeirão Preto, SP Brazil; 3 Department of Agriculture and Food Supply, Institute of Animal Science and Pastures of the São Paulo State, Nova Odessa, SP Brazil; 4 Department of Basic Sciences, School of Animal Science and Food Engineering, University of São Paulo, Pirassununga, SP Brazil; 5 Department of Animal Science, Luiz de Queiroz College of Agriculture, University of São Paulo, Piracicaba, SP Brazil

**Keywords:** Aflatoxin, Fumonisin, LAB, Yeast, Adsorption, Biomarker

## Abstract

Inactivated microorganisms may reduce mycotoxins’ bioavailability, although *in vivo* evidence is limited especially in farm animals. This pilot study evaluated whether a fermented milk (FM) containing heat-inactivated biomasses of *Lacticaseibacillus rhamnosus* and *Saccharomyces cerevisiae* at 1% (*w/w*) mitigated the toxic effects of dietary aflatoxins (AFs) and fumonisins (FBs) in weaned piglets. Twenty-four male crossbred piglets were assigned to a 2 × 2 × 2 factorial design (*n* = 3 per group) with AFs (0; 1.11–1.28 mg/kg, sum of AFB_1_, AFB_2_, AFG_1_ and AFG_2_), FBs (0.78–0.99; 9.22–9.53 mg/kg, sum of FB_1_ and FB_2_), and FM (0; 39 g/animal/day, equivalent to 5% of basal diet) for 28 days. AFs reduced (*P* < 0.05) the body weight gain and feed consumption. AFs or AFs + FBs exposure were associated with hepatic lesions, whereas FBs caused increased pulmonary and renal lesions (*P* < 0.05). FM was associated with lower (*P* < 0.05) scores of FBs’ lung lesions and AFs + FBs’ kidney lesions, but did not consistently improve growth performance or hepatic and renal biochemical parameters. Plasma AFP_1_ and urinary FB_2_ were lower (*P* < 0.05) in AFs- or FBs-exposed animals receiving FM, while plasma and urinary AFM_1_ or FB_1_ were not reduced by the FM. In conclusion, FM containing inactivated *L. rhamnosus* and *S. cerevisiae* had partial protective effects against organ-specific mycotoxin injury, with limited evidence of AFs bioavailability reduction, considering *n* = 3 per experimental group. Further studies should evaluate different inclusion levels of microorganisms and mycotoxins, as well as other potentially adsorptive microorganisms, with a focus on microorganism–mycotoxin interactions within the gastrointestinal tract.

**Supplementary Information:**

The online version contains supplementary material available at https://doi.org/10.1007/s12550-026-00677-7.

## Introduction

Mycotoxins are ubiquitous contaminants of food and feedstuffs, and their co-occurrence is a major challenge for human and animal health with consequent implications in food and feed safety. Among them, aflatoxins (AFs) and fumonisins (FBs) are particularly relevant because of their frequent occurrence in food and feed ingredients and their well-documented toxicity in animals (Niaz et al. 2025). Aflatoxin B_1_ (AFB_1_), B_2_ (AFB_2_), G_1_ (AFG_1_), and G_2_ (AFG_2_) are produced by some species of fungi from the *Aspergillus* genus, while fumonisins (FBs), likely fumonisin B_1_ (FB_1_) and fumonisin B_2_ (FB_2_), are *Fusarium* toxins. (Pleadin et al. 2019). Worldwide occurrence data demonstrated 27% of feed and feed raw materials are contaminated with AFs, with an average contamination level of 58 µg/kg (Streit et al. 2013). Among several types of foods, the average contamination by AFs is reported to be around 28%, with an average concentration of up to 63.28 µg/kg (Ali et al. 2023). Regarding FBs, a global survey exhibited 60% of occurrence in food, with median levels of 723 µg/kg (Gruber-Dorninger et al. 2019). In Brazil, analysis of finished feed commonly shows frequent AFs and FBs contamination, including their co-occurrence. This pattern is relevant for swine production because climate conditions in tropical and subtropical regions may favor the growth of toxigenic *Aspergillus* and *Fusarium* spp. during production, storage, and feed processing (Rodrigues et al. 2026). Studies have reported AFs in around 21 and 58.6% of broilers and cattle feed, respectively, while the frequencies of FBs were 87 and 40.8% in the same feed types, respectively (Kobashigawa et al. 2019; Biscoto et al. 2022), while higher co-occurrence percentage of 62.5% was reported by Pires et al. (2025).

The dietary exposure to mycotoxins can pose serious health issues not only to the animals but to humans through the consumption of animal-derived foods. For example, exposure to AF in food can led to hepatotoxicity, immunosuppression, teratogenicity, and mutagenicity both in the humans and animals (Cimbalo et al. 2020). After ingestion, AFB_1_ is biotransformed mainly in the liver through cytochrome P450 (CYP) dependent pathways, producing hydroxylated and demethylated metabolites such as aflatoxin M_1_ (AFM_1_), aflatoxin Q_1_ (AFQ_1_), and aflatoxin P_1_ (AFP_1_). Although AFB_1_ biotransformation pathways vary between different mammalian species, AFM_1_ and AFQ_1_ are among the major metabolites formed during its biotransformation in pigs (Rushing and Selim 2019). Due to their presence in milk, urine and blood plasma, these metabolites are considered useful biomarkers of AFB_1_ exposure, as they indicate its internal AFs dose (Gregorio et al. 2015; Rushing and Selim 2019). FBs can disrupt the sphingolipid metabolism in cell membranes by interfering with ceramide synthase activity, which alters the balance of sphinganine and sphingosine and contribute to cellular toxicity in humans and animals (Gelineau-van Waes 2022). Unlike AFB_1_, FBs undergo limited biotransformation after ingestion. Minor hydrolysis, transamination and acylation reactions may occur in the liver and gastrointestinal tract, but these pathways do not generate widely used exposure biomarkers. For this reason, quantifiable levels of parent compounds such as FB_1_ and FB_2_ in blood, plasma, and urine are considered viable biomarkers of FBs exposure (Shephard et al. 2007; Wang et al. 2016). The toxicological relevance of AFs and FBs is reflected in their carcinogenic classification. AF exposure is associated with hepatocellular carcinoma, supporting the classification of naturally occurring AFs as carcinogenic to humans, Group 1, by the International Agency for Research on Cancer (IARC, 2002; IARC, 2012). FBs are classified as Group 2B, possibly carcinogenic to humans, mainly because of the association between FBs exposure and esophageal cancer (IARC, 2002; Gelineau-van Waes 2022).

Pigs are highly sensitive to both AFs and FBs-induced toxicosis (Richard 2007; Di Gregorio et al. 2017a; Terciolo et al. 2019). In pigs, AFs intake is commonly associated with impaired growth, reduced feed efficiency, as well as hematological changes, serum biochemical alterations, and histopathological alterations mainly in the liver (Weaver et al. 2013; Di Gregorio et al. 2017a). FBs exposure may cause mild to moderate changes in hematological and serum biochemical parameters. However, its most consistent effects are histopathological alterations in several organs, particularly the lungs (Grenier et al. 2011; Souto et al. 2015; Terciolo et al. 2019). At high dietary levels, FBs-contaminated feed can cause porcine pulmonary edema (PPE), a severe toxicosis characterized by dyspnea, cyanosis, pulmonary edema, hydrothorax, and death in acute cases (Voss et al. 2007; Gao et al. 2023).

As mycotoxin contamination can occur at several stages of the food and feed production chain, good agricultural and post-harvest practices remain essential to reduce fungal growth and mycotoxins production. However, prevention alone may not fully eliminate exposure risk, because AFs and FBs are heat stable and can resist several conventional processing conditions (Khan et al. 2024; Shekhar et al. 2025; Kovač Tomas and Jurčević Šangut 2025). Therefore, mitigation strategies have been proposed to reduce the mycotoxin bioavailability after contaminated feed is ingested, including biological approaches for microbial binding/adsorption of mycotoxins in the gastrointestinal tract (Zhang et al. 2019; Ben Taheur et al. 2020), thus limiting their absorption and systemic exposure (Corassin et al. 2013; Avantaggiato et al. 2014; Campagnollo et al. 2015; Zhao et al. 2016). Several *in vitro* studies have shown that microorganisms, especially lactic acid bacteria (LAB) and yeasts, can bind AFs and FBs through interactions with cell-wall components (Niderkorn et al. 2007; Pizzolitto et al. 2012; Bovo et al. 2014; Ismail et al. 2017; Muaz et al. 2021; Pires et al. 2022, 2024). Under optimized *in vitro* conditions, thermally inactivated *Lacticaseibacillus rhamnosus*, *Saccharomyces cerevisiae* and their combinations, adsorbed 42.8, 84.8, and 91.2% of AFB_1_, respectively (Pires et al. 2024). For FBs, *L. rhamnosus* GG adsorbed up to 24% of FB_1_ and 72% of FB_2_. Similarly, *Lactobacillus delbrueckii* spp. *bulgaricus* adsorbed up to 31% of FB_1_ and 82% of FB_2_ (Niderkorn et al. 2006).

Several *in viv*o studies have evaluated the efficacy of mineral, microbial, or combined adsorbents to reduce mycotoxin toxicity in pigs, broilers, laying hens, buffaloes, goats, dairy cows, rats, and humans as experimental or target species. Common endpoints include growth performance, hematology, serum biochemistry, organ weight, histopathology, oxidative stress markers, and mycotoxin biomarkers as complementary data to the endpoints in adsorption studies (Robinson et al. 2012; Freire et al. 2022; Dionisio Freire et al. 2024). Biomarker-based approaches are especially useful because they provide evidence of internal exposure and evaluate individually whether adsorbents reduce their bioavailability (Di Gregorio et al. 2017a; Gonçalves et al. 2017; Souto et al. 2017; Freire et al. 2022; Aoyanagi et al. 2023; Ali et al. 2025). While the available* in vivo* studies have assessed the efficacy of chemical or organic adsorbents by mixing them directly to the feed, this approach is not feasible for testing microbial cells against mycotoxins due to uniform mixing challenges and possible structure degradation of cells caused by feed matrix interactions. In human nutrition, fermented dairy products are suitable carriers for microorganisms because they can incorporate their biomasses and deliver them directly to the gastrointestinal tract (Lucatto et al. 2020). However, information on the potential use of dairy matrices as carriers for mycotoxin-mitigating microbial agents remains scarce. Abdel-Salam et al. (2020) reported that a functional yogurt containing chicory root extract mitigated AFB_1_ toxicity in rats, with improvements in biochemical parameters and oxidative stress markers. However, to the authors’ knowledge, there is no information available on the protective effects of fermented dairy matrices containing inactivated microorganisms against the toxic effects of dietary mycotoxins in farm animals such as pigs. We therefore undertook a proof-of-concept study using a fermented milk (FM) as vehicle for inactivated biomasses of *L. rhamnosus* and *S. cerevisiae* to ameliorate the toxic effects of dietary AFs and FBs in weaned pigs.

## Materials and methods

### Preparation of lactic acid bacteria and yeast biomasses

The microbial biomasses used in this study were lyophilized commercial strains of *L. rhamnosus* (HOWARU^®^ Rhamnosus LYO 40 DCU, Danisco Ltd., São Paulo, Brazil) (LR) and *S. cerevisiae* (Fermentis K-97, SafAle, Bruggeman, Gent, Belgium) (SC). Both products had a declared cell concentration of 1.0 × 10^10^ cells/g. Before adding to the FM, LR and SC biomasses were thermally inactivated by autoclaving at 121 °C for 10 min. Heat treatment was used to inactivate the microorganisms and to modify cell-wall structures involved in mycotoxin binding, including peptidoglycan and β-D-glucans, as these structural changes may increase the availability of binding sites for mycotoxins (Haskard et al. 2000, 2001; Niderkorn et al. 2006). The combined efficacy of heat-inactivated LR and SC biomasses to adsorb AFB_1_ in the FM was previously assessed *in vitro* by Pires et al. (2024) at inclusion percentages of 0.5, 1.0, 2.0, and 4.0%, to adsorb AFB_1_ at 1.0 µg/g. The respective adsorption rates were 65.3, 91.2, 88.2, and 86.9%, while the FM containing 1.0–4.0% of heat-inactivated LR + SC reduced the AFB_1_ cytotoxicity in mouse embryonic fibroblasts (MEF-1).

## Preparation of the fermented milk

The FM was produced as described by Soukoulis et al. (2007), with modifications as reported by Pires et al. (2024), including the addition of LR and SC biomasses. Skim milk was obtained from a dairy plant and standardized by adding skim milk powder to reach 12.5% total solids content (TS, *w/w*). Triplicate samples (100 mL) of the standardized milk were analyzed for TS, fat, lactose, minerals, and total protein using a Lactoscan^®^ MCC analyzer (Milkotronic, Nova Zagora, Bulgaria), and the results were 12.46 ± 0.11, 0.10 ± 0.01, 5.92 ± 0.04, 0.78 ± 0.03 and 3.98 ± 0.03%, respectively. Milk samples were also analyzed in triplicate according to Jager et al. (2013), and found to have AFB_1_ or AFM_1_ at levels below the detection limits of the analytical method (0.1 and 0.075 µg/mL, respectively). The standardized milk was transferred to 15-L yogurt-producing vats, and heat-inactivated LR and SC were added at 1.0% LR + 1.0% SC (w/w), followed by mixing for 10 min. The milk containing LR and SC was heated-treated at 90 °C for 15 min, then reduced to 42 °C and followed by the inclusion of the starter culture containing *Streptococcus thermophilus* and *Lactobacillus delbrueckii* ssp. *bulgaricus* (Yo-Flex, Chr. Hansen, Horsholm, Denmark) at 3.0% inoculum. The mixture was incubated at 42 °C until pH 4.5 was reached, in approximately 3 h. The FM was cooled to 10 °C and transferred to polyethylene bottles. The bottles were stored at 5 °C for a maximum of 30 days until use. Triplicate samples (100 g) of the fermented milk were collected on day 1 of storage and subjected for analysis of physicochemical characteristics according to the Association of Official Analytical Chemists (Cunniff 1995). TS, fat, lactose and total protein percentages were 12.51 ± 0.10, 0.09 ± 0.01, 5.74 ± 0.03 and 3.83 ± 0.04%, respectively. Titratable acidity and pH values were 0.95% (expressed as a percentage of lactic acid equivalent) and 4.34 ± 0.02, respectively.

## Animals, diets and experimental design

Twenty-four weaned male crossbred piglets, approximately 23 days old (body weight = 6.6 ± 0.5 kg), were purchased from a commercial breeding center. Piglets were housed individually in adjustable cages in a closed shed equipped with fans to maintain suitable environmental conditions. The experiment was approved by the Animal Ethics Committee of the Institute of Animal Science and Pastures (IASP) of Nova Odessa (protocol nº 408–2023).

AFs-contaminated culture material was produced using *A. flavus* FML263 strain, which was kindly donated by the Food Microbiology Laboratory, School of Food Engineering, State University of Campinas, Brazil. This strain was isolated from peanut grains from São Paulo State and genetically characterized using an internal transcribed spacer locus. AFs production was conducted as described by Shotwell et al. (1966). Briefly, the *A. flavus* strain was placed inside a microbiological safety cabinet and inoculated onto potato dextrose agar (PDA) plates (Oxoid, Thermo Fisher Scientific, Hampshire, UK) and incubated at 25 °C until growth. Subsequently, 76-g aliquots of sterile rice were weighed in Erlenmeyer flasks, which were inoculated with small pieces of PDA containing the *A. flavus* strain. The inoculated flasks were placed in a horizontal shaker (Tecnal TE-140, Tecnal, Piracicaba, Brazil), which was kept at 250 rpm and incubated for 1 week in the dark at room temperature. After this period, the flasks were placed in a chemical hood, and 35–40 mL of chloroform was carefully added in each flask to stop the growth of the *A. flavus* strain. The flasks were shaken vigorously for a few seconds to distribute the chloroform and the mixtures were transferred onto stainless steel trays, where they were spread in 1-cm layers. The trays were kept in a forced air oven at 40 °C for 48 h until dryness. After drying, the rice culture material was milled and stored at 4 °C until use. FBs-culture culture material was produced using *F. verticillioides* grown on crushed corn. One hundred grams of corn was placed in 500-mL Erlenmeyer flasks, moistened with water to obtain water activity > 0.97, and autoclaved at 121 °C for 1 h. A fungal conidial suspension was prepared after culture on PDA for 15 days at 25 °C. Two milliliters of conidial suspension, containing 1 × 10^5^ conidia/mL, were inoculated into the autoclaved corn. The flasks were kept at 25 °C for 28 days. After incubation, the culture material was stored at 4 °C until use. AFs and FBs concentrations were determined in their respective culture materials according to Franco et al. (2019).

Two basal diets (BD) were used. During acclimatization, piglets received a pre-initial basal diet (P-BD) formulated with cornmeal (42%), soybean meal (18%), and 40% of pre-starter pig feed (essential additives for newly weaned piglets). During the experimental period, piglets received a BD composed of cornmeal (68%), soybean meal (28%), and starter pig feed (4%). Both diets were screened for basal mycotoxin contamination according to Franco et al. (2019), indicating non quantifiable levels of AFs and FBs levels varying from 0.78 to 0.99 mg/kg (sum of FB_1_ and FB_2_), as shown in Supplementary Table S1. The experimental BD was mixed with AFs- and/or FBs contaminated culture material using a “Y-type” mixer for 30 min. After mixing, triplicate samples of the contaminated BD were collected and analyzed according to Franco et al. (2019). The levels of AFs and FBs were selected based on previous studies that reported relevant signs of mycotoxicosis in pigs (Di Gregorio et al. 2017a; Souto et al. 2017; Terciolo et al. 2019). The mean measured levels in the experimental feed used in this study were 1.182 ± 0.780 mg/kg for total AFs (sum of AFB_1_, AFB_2_, AFG_1_ and AFG_2_), and 9.401 ± 1.428 mg/kg for total FBs (sum of FB_1_ and FB_2_) were. The individual AFs and FBs concentrations for each treatment group are provided in Supplementary Table S1. Moreover, low levels of naturally occurring zearalenone (ZEN) were found in the experimental feed (0.002 ± 0.0001 mg/kg), while T-2 toxin, ochratoxin A (OTA) and deoxynivalenol (DON) were not detected in any sample analyzed (detection limits: 0.0006, 0.0007, and 0.006 mg/kg, respectively) (Franco et al. 2019).

Piglets were acclimatized for 7-days with free access to non-contaminated feed (P-BD) and water *ad libitum*. After acclimatization, animals were assigned into eight homogeneous groups (*n* = 3 per treatment). The number of experimental animals in each treatment was defined according to the amount of culture materials containing AFs and FBs, and the number of individual cages available in the IASP. The mean body weight at allocation was 8.9 ± 0.5 Kg. The 28 days experimental period followed a 2 × 2 × 2 factorial design, corresponding to two levels of AFs, FBs, and FM, as follows: (G1) BD only; (G2) BD contaminated with 1.13 mg/kg AFs; (G3) BD contaminated with 9.49 mg/kg FBs; (G4) BD contaminated with 1.11 mg/kg AFs and 9.53 mg/kg FBs; (G5) BD in addition with FM (5% BD); (G6) BD in addition with 1.18 mg/kg AFs and FM (5% BD); (G7) BD in addition with 9.22 mg/kg FBs and FM (5% BD); (G8) BD in addition with 1.21 mg/kg AFs and 9.37 mg/kg FBs, and FM (5% BD). Animals had free access to water *ad libitum* and were fed twice daily according to their assigned treatment. In FM-supplemented groups, FM was provided 5 min before the experimental diets using stainless-steel feeders. Animals received 30 g/day FM during the first 14 days and 48 g/day during the last 14 days of experiment, which corresponded to approximately 5% of daily feed intake. Throughout acclimatization and the experimental periods, the animals were monitored daily by a qualified veterinarian and an assistant. Health status, environmental temperature, and hygiene conditions were recorded and controlled. Piglets were weighed weekly, and feed consumption measured weekly.

## Sample collection, biochemical and histological analyses

Blood samples were collected at the beginning of the experimental period (D0), and every 14 days (D14 and D28). Samples were obtained by aseptic jugular venipuncture using an evacuated blood-collection system. Blood samples were collected into 9 mL clot activator tubes (Vacuette Greiner Bio-One, Santa Bárbara D’Oeste, Brazil) and 6 mL sodium heparin tubes (Labor Import, Osasco, Brazil). Tubes were stored at 4 °C for approximately 2 h and then centrifuged at 1,610 × g for 10 min (Fanem Excelsa II mod. 206 BL, Guarulhos, Brazil). Serum and plasma supernatants were transferred to 15-mL polypropylene tubes and stored at − 20 °C until analysis.

Serum samples were used for biochemical analysis of creatinine (CREA), alanine aminotransferase (ALT), aspartate aminotransferase (AST), alkaline phosphatase (ALP), total protein (TP), and albumin (ALB). Analyses were performed using an automated Mindray BS-120 biochemistry analyzer (Shenzhen, China) and commercial kits (Labtest, Lagoa Santa, Brazil).

Urine samples were collected at the beginning of the experimental period and every 7 days thereafter, using pediatric urine collectors (Labor Import, Osasco, Brazil). Before each collection, the genital area of each piglet was gently cleaned using warm water and mild soap, then dried completely with disposable paper tissues. After removing the paper lid from the adhesive part of the bag, it was carefully positioned over the piglet’s penis. The piglets were kept under observation and, immediately after urination, the bag was removed and the urine was transferred into sterile 100-mL urine containers. Urine samples were stored at − 20 °C until analysis. Urinary creatinine was measured using an automated Mindray BS-200 biochemistry analyzer (Mindray, Shenzhen, China) and a commercial kit (Bioclin, Belo Horizonte, Brazil).

At the end of the experiment, the animals were stunned by electronarcosis and euthanized by exsanguination. Samples of heart, liver, lung, kidney, spleen, jejunum, colon, and mesenteric lymph nodes were collected and individually placed in sterile polypropylene flasks. Each flask was labelled with the tissue name and the code of its respective treatment. Tissue samples were fixed in 10% buffered formalin 10% (Sigma-Aldrich, St. Louis, USA) and 70% ethyl alcohol. For histological assessment, tissue sections were stained with hematoxylin and eosin and evaluated according to Neeff et al. (2013). Microscopic lesions were assessed by a certified veterinary pathologist who was not aware about the treatments’ codes. Each sample was scored from 0 to 3 according to severity, with score 0 indicating no relevant microscopic lesion. Scores 1, 2 and 3 indicated mild lesions affecting up to 25%, 26–50% and more than 51% of the evaluated section, respectively.

## Analyses of aflatoxins and fumonisins in plasma and urine

The reagents used for sample preparation and chromatographic analysis included HPLC-grade formic acid (JT. Baker, Xalostoc, Mexico), acetonitrile, methanol and acetic acid, which were purchased from Sigma-Aldrich (Burlington, USA). Milli-Q water was produced by Millipore Academic (Millipore Corporation, Billerica, USA). Analytical standards of AFM_1_, AFP_1_, AFQ_1_, FB_1_, and FB_2_ were obtained from Sigma-Aldrich (St. Louis, USA). Isotopically labeled internal standards [¹³C₁₇]-AFM_1_ and [¹³C₃₄]-FB_1_ used were obtained from Biopure, Romer Labs (Tulln, Austria).

Plasma samples were prepared for chromatographic analysis following the methodology of Muñoz-Solano and González-Peñas (2023) with some modifications. In brief, 450 µL plasma was mixed with 50 µL of β-glucuronidase/arylsulfatase type H-3 from *Helix pomatia* (Sigma-Aldrich, Cotia, Brazil) in a 2-mL microcentrifuge tube. The mixture was incubated overnight at 37 °C. After incubation, the solution was transferred to a 3-mL syringe coupled to a Captiva EMR-Lipid column (Agilent Technologies, Barueri, Brazil). Then, 1.5 mL of acetonitrile containing 1% formic acid was added. After 5 min, the solution was passed through the column using a syringe plunger. The microcentrifuge tube was washed with 500 µL of acetonitrile/water (75:25, *v/v*) containing 1% formic acid. This wash solution was also passed through the Captiva column. The eluate was evaporated to dryness at 60 °C under nitrogen flow at 0.13 bar using a MultiVap-54 system (LabTech, Sorisole, Italy). The residue was reconstituted in 450 µL of acetonitrile/water (10:90, *v/v*), vortexed, filtered through a 0.22-µm PTFE syringe filters (Perfecta, São Paulo, Brazil), and stored at − 4 °C until analysis.

Urine samples were prepared according to Ali et al. (2025) with minor modifications. Briefly, 1 mL of urine was centrifuged at 7300 × g for 5 min (Sigma Laborzentrifugen, Osterode am Harz, Germany), and 800 µL of the supernatant was mixed with 3 µL [¹³C₁₇]-AFM_1_ and 3 µL [¹³C₃₄]-FB_1_, reaching a final IS concentration of 1.9 ng/mL for each standard. Next, 800 µL of acetonitrile and 80 µL of acetic acid were added. The mixture was vortexed and centrifuged at 7300 × g for 5 min (Sigma Laborzentrifugen, Osterode am Harz, Germany). The cleanup step was performed using MycoSpin 400^®^ columns (Romer Labs, Tulln, Austria). In each run, 840 µL of the solution was transferred to the column, vortexed for 1 min, and centrifuged at 7300 × g for 5 min (Sigma Laborzentrifugen, Osterode am Harz, Germany). This procedure was repeated twice. The total filtered volume, 1.68 mL, was transferred to vials and dried at 45 °C under nitrogen flow at 0.13 bar using a MultiVap-54 system (LabTech, Sorisole, Italy). The dried extracts were reconstituted in 400 µL methanol/water (50:50, *v/v*), filtered through 0.22-µm PTFE syringe filters (Perfecta, São Paulo, Brazil), and stored at − 4 °C until analysis.

### Liquid chromatography and mass spectrometry conditions

Plasma and urine extracts were analyzed using an ultra-performance liquid chromatographic (UPLC) system Waters Acquity I-Class (Waters, Milford, USA) with a BEH C18 column (2.1 × 50 mm, 1.7 μm) and coupled with a triple quadrupole mass spectrometer (MS/MS) Xevo TQ-S^®^ (Waters, Milford, USA). Five microliters of the plasma and urine extracts as well as standards were injected into the UPLC-MS/MS system. Chromatographic conditions, including column and temperature, mobile phase, solvents, elution, and flow rate, were exactly as described by Franco et al. (2019) and displayed in Supplementary Table S2. For plasma biomarkers, 6-point matrix-matched calibration curves were prepared at concentrations of 0.16, 0.31, 0.62, 1.24, 2.48, and 45.0 ng/mL for AFM_1_, AFQ_1_, AFP_1_, and 0.39, 1.55, 3.09, 6.19, 12.38, 225.0 ng/mL FB_1_ and FB_2_. For urine biomarkers, the 6-point matrix-matched calibration curves had concentrations of 0.16, 0.14, 0.62, 1.25, 2.5, 5.0 ng/mL for AFM_1_, AFQ_1_, AFP_1_, and 3.12, 6.25, 12.5, 25.0, 50.0, 100.00 ng/mL for FB_1_ and FB_2_. As urine samples were prepared by adding IS standards before extraction, these IS were used to normalize sample responses, which compensated for both recovery losses during sample processing and matrix effects.

The mass spectrometer was operated in multiple reaction monitoring mode using positive electrospray ionization. The MS/MS parameters followed Franco et al. (2019): cone gas flow, 150 L/h; desolvation gas flow, 800 L/h; desolvation temperature, 500 °C; source temperature, 150 °C; and capillary voltage, 3 kV. Cone voltage, collision energy, and MRM transitions were optimized for each analyte, as shown in Supplementary Table S3. Limits of detection and quantification were calculated using signal-to-noise ratios of 3:1 and 10:1, respectively. Data acquisition and processing were performed using MassLynx version 4.1.

## Performance evaluation of the analytical method for mycotoxins in plasma

The performance of the analytical method for determination of mycotoxins in plasma samples was evaluated using blank pig plasma, as described by Di Gregorio et al. (2017b), with minor amendments. A pooled plasma sample from piglets fed only BD was fortified in triplicate with AFM_1_, AFQ_1_ and AFP_1_ at concentrations of 0.16, 0.31, 0.62, 1.24, 2.48, 45.0 ng/mL, and with FB_1_ and FB_2_ at concentrations of 0.39, 1.55, 3.09, 6.19, 12.38, 225.0 ng/mL. Fortified samples were prepared and analyzed using the same extraction and UPLC-MS/MS procedures as described above.

For matrix-effect and recovery evaluation, the same mycotoxin concentrations were added to extracts prepared from non-fortified pooled plasma and to acetonitrile/water (10:90, *v/v*). Matrix-matched calibration curves were prepared from these data. The signal suppression/enhancement (SSE) due to matrix effects, recovery of extraction (*R*_E_), and apparent recovery (*R*_A_) were calculated according to Eqs. 1, 2 and 3, respectively as described by Sulyok et al. (2006). In the equations, *Sse* represents the slope of the spiked extract, *Ssp* the slope of the spiked sample, and *Sls* the slope of the liquid standard (Sulyok et al. 2006). The concentration of each mycotoxin in plasma samples was corrected for recovery. The limits of detection (LOD) and quantification (LOQ) in all analyses were calculated using signal-to-noise ratios of 3:1 and 10:1, respectively.

1 $$SSE\left(\%\right)=\left(\frac{Sse}{Sls}\right)\:\times\:100$$

2 $$RE\left(\%\right)=\left(\frac{RA}{SSE}\right)\:\times\:100$$

3 $$\:RA\left(\%\right)=\left(\frac{Ssp}{Sls}\right)\:\times\:\:100$$

### Data analysis

Growth performance and serum biochemistry data were subjected to ANOVA as a 2 × 2 × 2 factorial using the GLM procedure in SAS software. Variable means for treatments showing significant differences in the ANOVA were compared using the Tukey-Kramer’s procedure. Analytical results below the LOQ of mycotoxin biomarkers in plasma and urine samples were adjusted using the LOQ/2 approach described by Keizer et al. (2015). Given the absence of normality of data on plasma and urine biomarker concentrations as well as histopathological scores, results were analyzed using the Kruskal–Wallis test followed by Dunn’s multiple-comparison test. These analyses were performed in R software, version 4.5.1. Statistical significance was set at *P* < 0.05.

## Results and discussion

### Growth performance

No mortality was observed during the 28-day experimental period. However, some animals showed clinical signs compatible with mycotoxicosis. Piglets from control group (G1) and G3 (FBs alone) presented apathy, emesis, and hyporexia for a few days. Animals from G2 (AFs alone), G4 (AFs + FBs), and G8 (AFs + FBs and FM) showed apathy and hyporexia. One animal from G7 (FBs and FM) presented diarrhea for most of the experimental period. More frequent coughing was observed in G2 (AFs alone), G3 (FBs alone), and G4 (AFs + FBs) than in other groups. Rooting behavior was frequent and caused feed wastage, which may have reduced the precision of feed-consumption estimates.

The effects of the treatments on animal performance are presented in Table 1. AFs exposure significantly decreased body weight gain (BWG) (*P* = 0.003) and feed consumption (FC) (*P* = 0.002) in piglets over 28 days of intoxication, with significant main effects detailed in Supplementary Table S4. However, no significant interactions (*P* > 0.05) between AFs, FBs, and FM were observed for BWG, FC or F : G. The negative effect of AFs on performance agrees with those reported by Di Gregorio et al. (2017a), who observed that pigs fed an AFB_1_ contaminated diet at 1.1 mg/kg for 21 days exhibited reduced BWG, average daily gain, average daily feed intake, and gain: feed ratio. Aoyanagi et al. (2023) also reported reduced BWG in piglets fed diets containing AFs (0.5 mg/kg), FBs (1.0 mg/kg), and ZEN (1.0 mg/kg) for 42 days, although FC and F : G were not affected. Holanda et al. (2020) reported that a yeast cell wall-based adsorbent did not ameliorate the negative effects caused by dietary AFs (2.0 mg/kg) and DON (0.2 mg/kg) on BWG, average daily gain (ADG), and average daily feed intake (ADFI) of piglets during 36 days, which is consistent with the limited effect of FM on growth performance observed in the present work.

**Table 1:** Efficacy of a functional fermented milk (FM) to ameliorate the toxic effects of individual and combined dietary exposure to aflatoxins (AFs) and fumonisins (FBs) on performance of piglets over 28 days of intoxication^1^

Treatment group	AFs^2^ (mg/kg)	FBs^3^ (mg/kg)	FM^4^ (g/animal/day)	Body weight gain (kg)	Feed consumption (kg)	Feed : gain
G1 (Basal diet)	< 0.01 ^5^	0.96	0	13.90 ± 2.04	22.82 ± 1.83	1.66 ± 0.16
G2	1.13	0.96	0	11.97 ± 2.78	21.51 ± 2.36	1.83 ± 0.23
G3	< 0.01 ^5^	9.49	0	13.20 ± 3.75	21.90 ± 3.90	1.70 ± 0.22
G4	1.11	9.53	0	8.453 ± 2.82	16.08 ± 4.05	1.95 ± 0.26
G5	< 0.01 ^5^	0.99	39 ^6^	15.04 ± 1.17	23.56 ± 0.62	1.57 ± 0.12
G6	1.28	0.78	39^6^	11.02 ± 3.53	18.99 ± 5.01	1.75 ± 0.12
G7	< 0.01 ^5^	9.22	39 ^6^	14.70 ± 0.97	25.31 ± 0.44	1.73 ± 0.13
G8	1.21	9.37	39^6^	10.91 ± 0.50	18.40 ± 2.94	1.68 ± 0.21
Source of variation				------------------------------ *P*-value ----------------------------		
AFs				0.003	0.002	0.094
FBs				0.270	0.313	0.439
FM				0.326	0.441	0.202
AFs x FBs				0.536	0.189	0.631
AFs x FM				0.789	0.396	0.358
FBs x FM				0.371	0.152	0.826
AFs x FBs x FM				0.468	0.670	0.349

^1^ Data are means ± SD of 3 piglets per treatment evaluated at 7, 14, 21 and 28 days of intoxication

^2^ Sum of aflatoxins B_1_, B_2_, G_1_ and G_2_

^3^ Sum of fumonisins B_1_ and B_2_

^4^ Fermented milk containing 1.0% (*w*/*w*) of cell biomasses of *Lacticaseibacillus rhamnosus* (1.0 × 10^10^ cells/g) and *Saccharomyces cerevisiae* (1.0 × 10^10^ cells/g) inactivated by autoclaving at 121 °C for 10 min

^5^ Limit of quantification for each aflatoxin

^6^ Equivalent to approximately 5% of the daily feed intake

### Serum biochemistry parameters

The effects of treatments on serum biochemical parameters are presented in Table 2. Treatment-related changes were observed for ALT, ALP, ALB and creatinine levels. No significant effects of individual factors or their interactions were observed for AST and TP (*P* > 0.05). However, ALP levels were significantly higher in AFs-exposed animals (*P* = 0.003) and receiving FM (*P* = 0.019), with no significant interaction effects among factors (*P* > 0.05). For ALT levels, interaction effects between AFs × FBs (*P* = 0.001) and AFs × FM (*P* = 0.033) were found, indicating that ALT responses depended on the combination of treatments, as further detailed in Supplementary Table S5. In particular, higher (*P* < 0.05) ALT levels were observed in animals exposed to both AFs and FBs, highlighting a combined effect of these mycotoxins to induce hepatic damage. Additionally, the interaction between AFs × FM indicate that AFs increased ALT levels only in the absence of FM, whereas no differences were observed when FM was administered, due to overall elevated ALT values (Supplementary Table S5). This finding suggests that FM altered the response to AF exposure, although without reducing enzyme levels.

**Table 2:** Efficacy of a functional fermented milk (FM) to ameliorate the toxic effects of individual and combined dietary exposure to aflatoxins (AFs) and fumonisins (FBs) on serum biochemistry of piglets over 28 days of intoxication ^1^

Treatment group	AFs^2^ (mg/kg)	FBs^3^ (mg/kg)	FM^4^ (g/animal/day)	ALT (U/L)	AST (U/L)	ALP (U/L)	TP (g/dL)	ALB (g/dL)	Creatinine (mg/dL)
G1 (Basal diet)	< 0.01 ^5^	0.96	0	85.56 ± 22.19	93.44 ± 37.51	539.8 ± 238.2	5.034 ± 0.479	2.923 ± 0.289	0.654 ± 0.071
G2	1.13	0.96	0	86.89 ± 12.00	91.78 ± 31.31	803.2 ± 109.3	5.113 ± 0.508	3.056 ± 0.307	0.719 ± 0.067
G3	< 0.01 ^5^	9.49	0	78.33 ± 23.53	72.89 ± 38.08	645.1 ± 302.9	5.124 ± 0.587	2.877 ± 0.405	0.630 ± 0.075
G4	1.11	9.53	0	131.6 ± 35.05	111.0 ± 42.12	735.4 ± 229.5	4.862 ± 0.163	2.674 ± 0.119	0.579 ± 0.058
G5	< 0.01 ^5^	0.99	39 ^6^	111.7 ± 37.76	73.56 ± 23.24	773.3 ± 232.8	4.848 ± 0.399	3.028 ± 0.408	0.639 ± 0.072
G6	1.28	0.78	39 ^6^	94.56 ± 29.71	80.11 ± 21.76	843.1 ± 226.0	5.247 ± 0.366	3.160 ± 0.179	0.649 ± 0.068
G7	< 0.01 ^5^	9.22	39 ^6^	106.9 ± 17.72	93.78 ± 39.27	696.0 ± 207.1	5.014 ± 0.457	2.898 ± 0.352	0.676 ± 0.062
G8	1.21	9.37	39 ^6^	122.8 ± 28.98	83.22 ± 18.36	986.7 ± 511.4	5.036 ± 0.228	2.938 ± 0.161	0.611 ± 0.048
Source of variation				------------------------------------------------------------- *P*-value ------------------------------------------------------------					
AFs				0.041	0.295	0.003	0.552	0.716	0.509
FBs				0.020	0.477	0.948	0.606	0.007	0.009
FM				0.040	0.216	0.019	0.980	0.083	0.900
AFs x FBs				0.001	0.464	0.826	0.074	0.132	0.003
AFs x FM				0.033	0.193	0.382	0.133	0.389	0.278
FBs x FM				0.586	0.425	0.902	0.769	0.788	0.010
AFs x FBs x FM				0.463	0.069	0.166	0.926	0.389	0.509

^1^ Data are means ± SD of 3 piglets per treatment evaluated at 0, 14 and 28 days of intoxication

^2^ Sum of aflatoxins B_1_, B_2_, G_1_ and G_2_

^3^ Sum of fumonisins B_1_ and B_2_

^4^ Fermented milk containing 1.0% (*w*/*w*) of cell biomasses of *Lacticaseibacillus rhamnosus* (1.0 × 10^10^ cells/g) and *Saccharomyces cerevisiae* (1.0 × 10^10^ cells/g) inactivated by autoclaving at 121 °C for 10 min

^5^ Limit of quantification for each aflatoxin

^6^ Equivalent to approximately 5% of the daily feed intake

*ALT* alanine aminotransferase,* AST* aspartate aminotransferase, *ALP* alkaline phosphatase, *TP* total protein, *ALB* albumin

The association of FM with increased levels of ALT and ALP was unexpected, as these enzymes were not affected in other animal models such as male Wistar rats receiving 5% of yogurt supplementation for 56 days (Lasker et al. 2019). Although there are no previous data on yogurt consumption by farm animals, Zhu et al. (2017) observed increased serum ALP levels in piglets receiving fermented feed ingredients (FFI). The authors attributed this effect to the improved digestive and absorptive functions provided by the FFI. Thus, the fact that piglets receiving FM alone (G5) in the present study had increased ALT and ALP levels without any sign of histopathological alterations may reflect subtle metabolic adaptations to the additional nutrient intake provided by the microbial biomass in the FM, rather than hepatocellular injury. Previous studies have reported variable effects of mycotoxins on hepatic enzymes, with some of them indicating that ALT was not increased in animals exposed to AFs, FBs, ZEN, OTA, DON, T-2 toxin (Pappas et al. 2014; Di Gregorio et al. 2017a; Aoyanagi et al. 2023; Fochesato et al. 2024; Vieira et al. 2024). In contrast, increased ALP has been reported in pigs fed AFB_1_-contaminated diets (Thieu et al. 2008). Di Gregorio et al. (2017a) also reported increased ALP in pigs exposed to AFB_1_, and found that hydrated sodium calcium aluminosilicate (HSCAS) did not prevent this increase. These findings are consistent with the persistence of ALP changes in the present study.

ALB was significantly affected by FBs (*P* = 0.007) (Table 2), with lower values observed in FBs-exposed animals as detailed in Supplementary **Table S5**. Such finding suggests that FBs exposure may affect hepatic synthetic activity or protein metabolism. However, this interpretation should remain cautious because ALB can also be influenced by nutrition, inflammation, hydration, and growth status. Previous studies have reported inconsistent effects of mycotoxins on ALB. Bovo et al. (2015) reported reduced ALB in broilers exposed to AFB_1_ (2.0 mg/kg), which was mitigated by the dietary inclusion of 1% beer fermentation residue containing *S. cerevisiae*. Deepthi et al. (2017) reported increased ALB in broilers fed FB_1_ (200.0 mg/kg) for 42 days, while supplementation with *L. plantarum* MYS6 restored ALB to control values. In pigs, Aoyanagi et al. (2023) found no differences in ALB among animals fed multiple mycotoxins and commercial adsorbents. Di Gregorio et al. (2017a) reported that a mineral adsorbent increased the serum ALB concentrations, although AFB_1_ itself did not directly affect this variable. These differences show that ALB responses may depend on the animal species, mycotoxin profile, exposure level, and adsorbent type.

Creatinine exhibited significant interactions between AFs × FBs (*P* = 0.003) and FBs × FM (*P* = 0.010) (Table 2), indicating that creatinine levels depended on the combination of treatments (Supplementary **Table S5**). The AFs × FBs interaction indicates that creatinine was reduced only in piglets co-exposed to AFs and FBs, while the FBs × FM interaction suggests that this parameter was reduced in FBs-exposed piglets only when FM was absent. However, reduced creatinine should not be interpreted alone as evidence of renal damage. Serum creatinine may be affected by body mass, growth rate, hydration, feed intake, and muscle metabolism. Therefore, renal effects as observed in the present study are better supported by kidney histopathology than by creatinine alone.Holanda et al. (2020) also reported reduced creatinine in pigs exposed to combined AFs and DON, while the tested adsorbent did not restore these values. Conversely, Grenier et al. (2011) reported increased creatinine in pigs fed an FB-contaminated diet. These contrasting findings suggest that creatinine responses may depend on the toxin combination, exposure period, animal age, and severity of renal injury. In summary, the results for ALT and creatinine indicate that co-exposure to AFs and FBs leads to distinct alterations in hepatic and renal biomarkers (Orsi et al. 2007), reinforcing the importance of considering combined mycotoxin exposure when evaluating toxicological effects. Considering the low number of replications per treatment used in this study, the protective effects of FM on biochemical parameters were limited and did not consistently counteract the alterations induced by mycotoxins.

### Histopathology

Histopathological findings are presented in Table 3. No relevant microscopic changes were observed in the heart, spleen, colon, or mesenteric lymph nodes. In control group (G1), mild lesions were restricted to the kidney and jejunum in some animals, which were not associated with clinical disease. Following these findings, Souto et al. (2015) also reported no cardiac alterations in piglets fed FB_1_-contaminated diets at 3.0, 6.0, or 9.0 mg/kg. In contrast, Terciolo et al. (2019) observed cardiac lesions in piglets fed 3.7 mg/kg FBs, including focal myocardial hemorrhage, lymphocytic inflammatory infiltrate, and multifocal cytoplasmic vacuolation of myocytes. The same study reported lymphoid alterations in mesenteric lymph nodes and spleen at 12.2 mg/kg FBs. These contrasting findings indicate that organ involvement may vary with FB dose, exposure duration, and individual susceptibility.

**Table 3:** Efficacy of a functional fermented milk (FM) to ameliorate the toxic effects of individual and combined dietary exposure to aflatoxins (AFs) and fumonisins (FBs) on histopathological changes in piglets after 28 days of intoxication ^1^

Treatment group	AFs^2^ (mg/kg)	FBs^3^ (mg/kg)	FM^4^ (g/animal/day)	Lung Median (Range)	Liver Median (Range)	Kidney Median (Range)	Jejunum Median (Range)
G1 (Basal diet)	< 0.01 ^5^	0.96	0	0 (0–0) ^b^	0 (0–0) ^b^	0 (0–1) ^bc^	0 (0–1) ^ab^
G2	1.13	0.96	0	2 (0–3) ^ab^	2 (1–2) ^a^	1 (0–1) ^abc^	2 (0–2) ^a^
G3	< 0.01 ^5^	9.49	0	3 (1–3) ^a^	0 (0–0) ^b^	2 (1–2) ^a^	1 (1–2) ^a^
G4	1.11	9.53	0	0 (0–2) ^ab^	2 (1–2) ^a^	1 (1–2) ^ab^	0 (0–1) ^ab^
G5	< 0.01 ^5^	0.99	39 ^6^	0 (0–0) ^b^	0 (0–0) ^b^	0 (0–0) ^c^	0 (0–0) ^b^
G6	1.28	0.78	39 ^6^	1 (1–1) ^ab^	0 (0–1) ^ab^	1 (1–1) ^abc^	1 (0–1) ^ab^
G7	< 0.01 ^5^	9.22	39 ^6^	0 (0–0) ^b^	0 (0–0) ^b^	2 (1–2) ^a^	0 (0–0) ^b^
G8	1.21	9.37	39 ^6^	0 (0–0) ^b^	1 (0–1) ^ab^	0 (0–0) ^c^	0 (0–1) ^ab^
*P*-value				0.023	0.009	0.014	0.145

^a−c^ Values within each column with no common superscript differ significantly by the Kruskal-Wallis test (*P* < 0.05)

^1^ Data are presented as medians and ranges (Q1–Q3) of 3 piglets per treatment, considering the results obtained after 28 days of intoxication

^2^ Sum of aflatoxins B_1_, B_2_, G_1_ and G_2_

^3^ Sum of fumonisins B_1_ and B_2_

^4^ Fermented milk containing 1.0% (*w*/*w*) of cell biomasses of *Lacticaseibacillus rhamnosus* (1.0 × 10^10^ cells/g) and *Saccharomyces cerevisiae* (1.0 × 10^10^ cells/g) inactivated by autoclaving at 121 °C for 10 min

^5^ Limit of quantification for each aflatoxin

^6^ Equivalent to approximately 5% of the daily feed intake

**Fig. 1:**
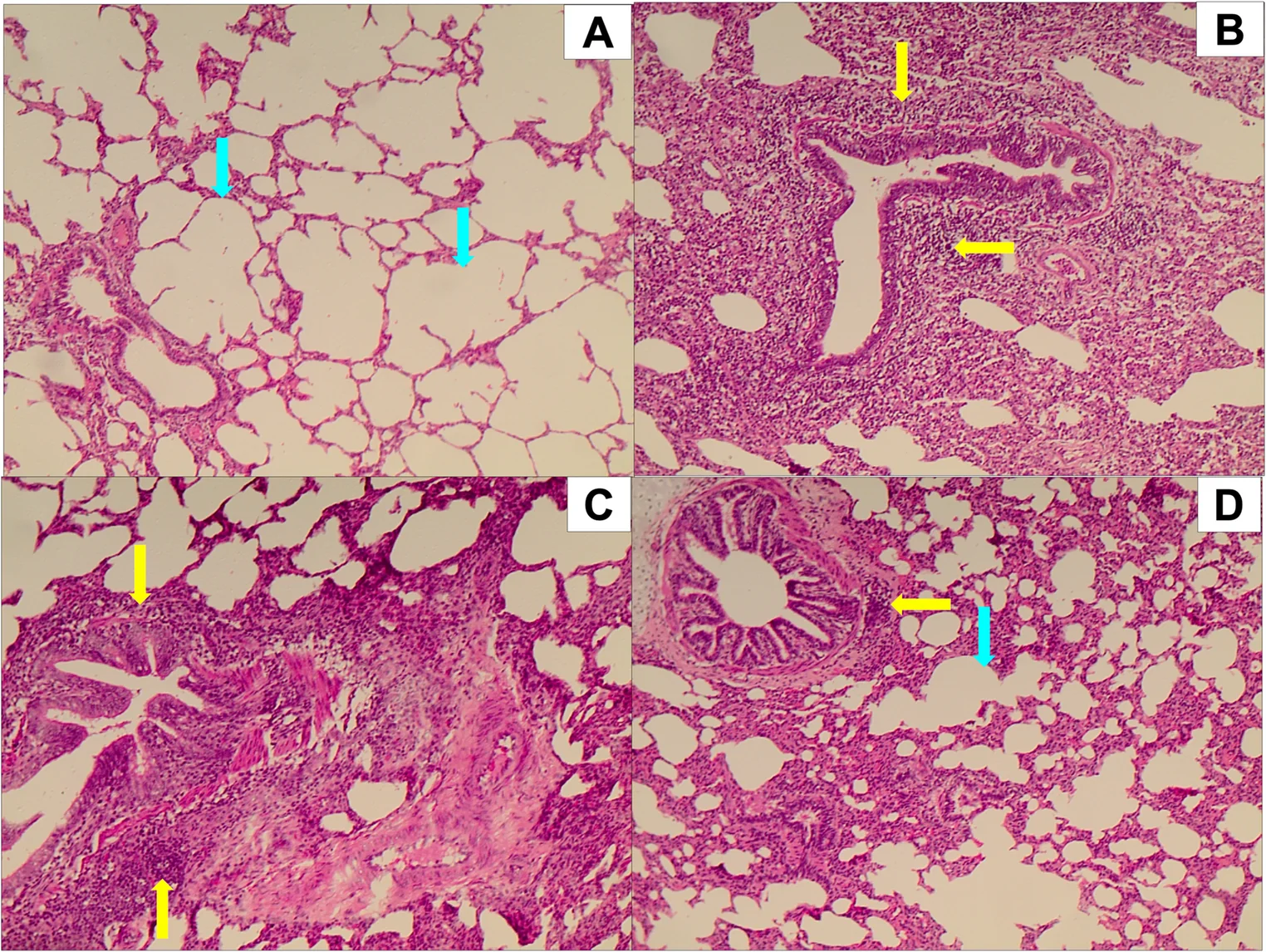
Representative microscopy photographs of hematoxylin- and eosin-stained lung sections (**A**–**D**), representing score 0 (**A**); score 3 (**B**); score 2 (**C**); score 1 (**D**). Yellow arrows: inflammatory infiltrate; Blue arrows: preserved alveoli. 200× magnification

In the lung, animals from G3 (FBs) showed the highest (severe) lesions score, which were characterized by intense inflammatory infiltrate and were significantly different (*P* < 0.05) from controls (G1). A representative image of lung with score 3 is shown in Fig. 1B, whereas Fig. 1A represents score 0. Lung lesions in G2 and G4 ranged from absent to severe (Fig. 1C and D), and were not significantly different from G1 (*P* > 0.05). FM supplementation was associated with lower (*P* < 0.05) pulmonary lesion scores in G7 (FBs + FM) and G8 (AFs + FBs + FM), compared with G3 (FBs) (Fig. 1A), thus suggesting that FM reduced FB-associated lung lesion severity rather than promoting a complete reversal. Pulmonary injury is a consistent target of FBs toxicity in pigs. Previous studies reported alveolar hemorrhage and edema, diffuse interstitial pneumonia (Terciolo et al. 2019), peribronchiolar hemorrhage, and alveolar edema in pigs exposed to FBs (Grenier et al. 2011). Aoyanagi et al. (2023) also reported that mineral adsorbents reduced the moderate interstitial pneumonitis in pigs fed diets contaminated with AFs, FBs, and zearalenone. In the present study, pulmonary edema was not observed. Notably, although pulmonary edema is a common and characteristic feature of FBs-mediated toxicity, it was not observed in the present study. However, the protective effect of FM against FBs-induced lung damage raises the possibility that it may also prevent pulmonary edema.

**Fig. 2:**
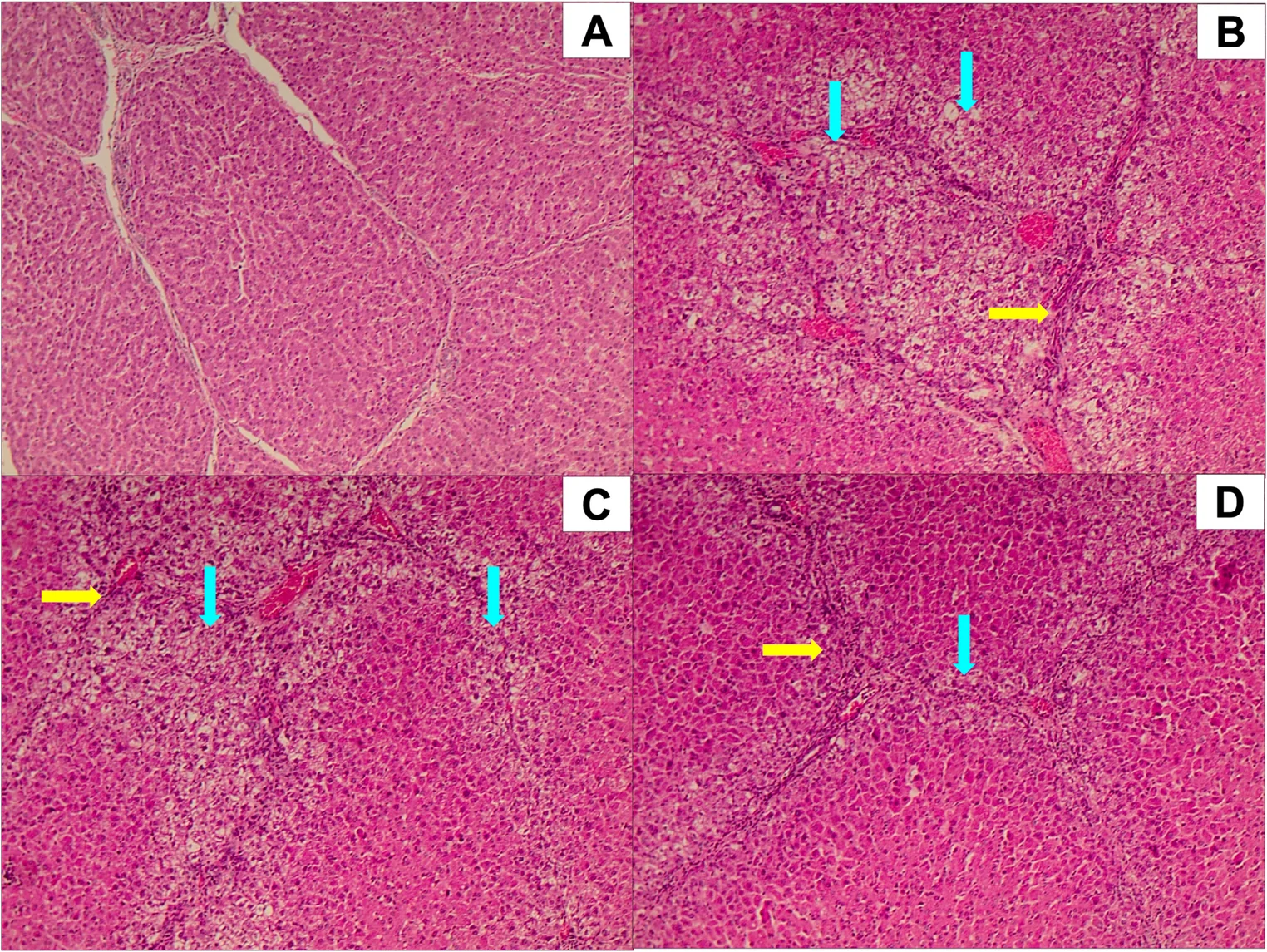
Representative microscopy photographs of hematoxylin- and eosin-stained liver sections (**A**–**D**), representing score 0 (**A**); score 2 (**B**); score 2 (**C**); score 1(**D**). Yellow arrows: inflammatory infiltrate; Blue arrows=hepatocellular ballooning. 200× magnification

In the liver, G2 (AFs) and G4 (AFs + FBs) showed mild to moderate lesions characterized by inflammatory infiltrate and hepatocellular ballooning, and these groups differed (*P* < 0.05) from controls (G1) and G3 (FBs). Representative liver sections are shown in Fig. 2, where Fig. 2A represents score 0 and Fig. 2B, C and D represents scores 3, 2, 1, respectively. FM did not significantly reduce (*P* > 0.05) hepatic lesion scores in AFs- or AFs + FBs-exposed piglets (G6 and G8, respectively). However, only one animal in G6 (AFs + FM) and two in G8 (AFs + FBs + FM) exhibited mild lesions, suggesting a trend toward reduced lesion severity, although this effect was not significant (*P* > 0.05). The hepatic lesions observed in AFs-exposed piglets agree with previous reports. Weaver et al. (2013) observed hepatic changes, including bile duct hyperplasia, hydropic degeneration, and karyomegaly in pigs fed a diet contaminated with AFs (0.15 mg/kg) and DON (1.1 mg/kg) for 42 days. Some commercial adsorbents reduced these hepatic changes. Studies on FBs associated liver damage are less consistent. Souto et al. (2015) reported no hepatic alterations in pigs fed up to 9.0 mg/kg FBs. Grenier et al. (2011) demonstrated hepatic damage at 6.0 mg/kg FBs. Terciolo et al. (2019) showed dose-dependent liver changes, with lesions occurring at 12.1 mg/kg FBs but not at 3.7 or 8.1 mg/kg. In the present study, FBs alone did not induce clear hepatic lesions. This suggests that the FBs level used here may have been insufficient to induce hepatic lesions, affecting especially the lung and kidney.

**Fig. 3:**
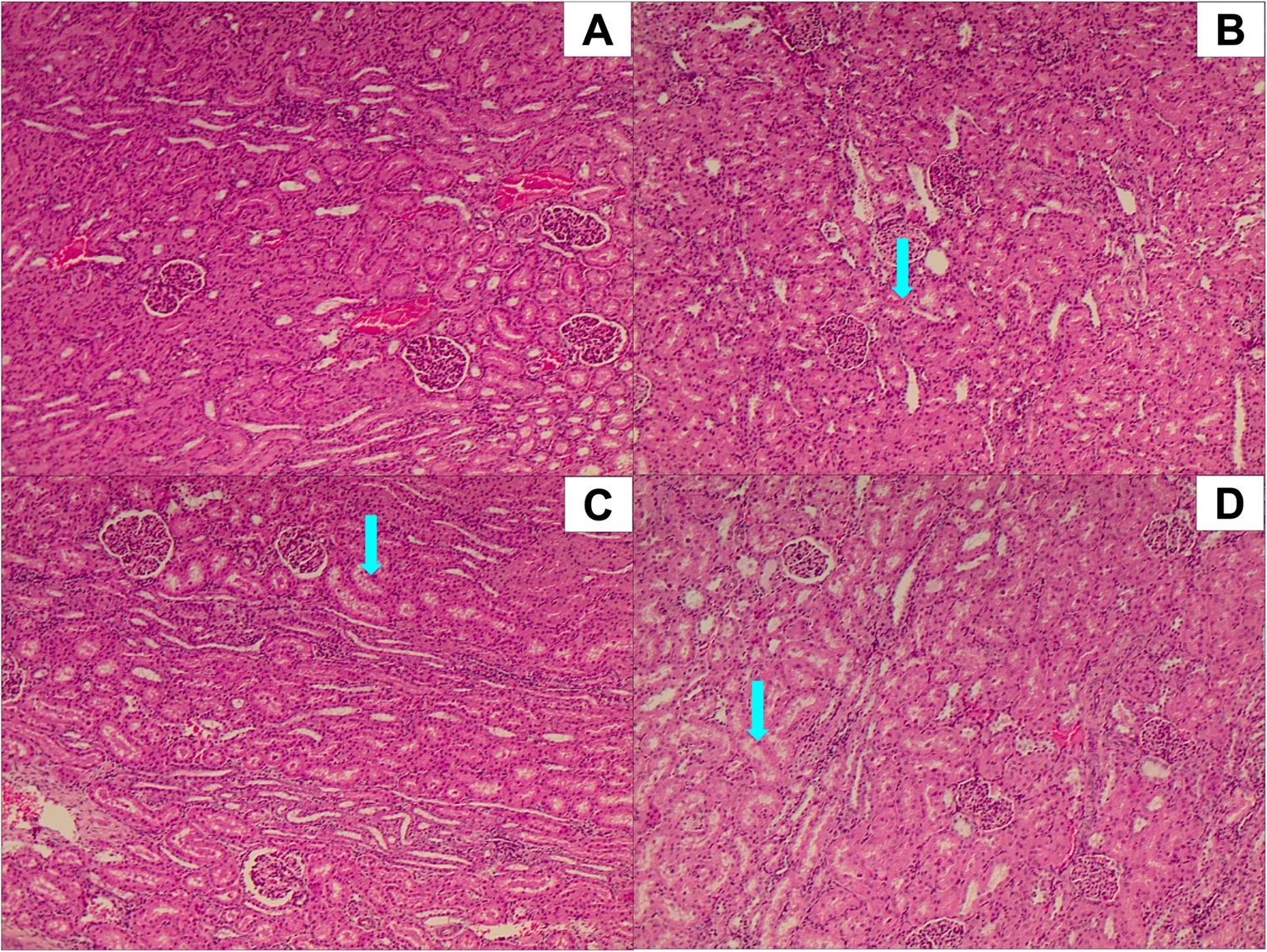
Representative microscopy photographs of hematoxylin- and eosin-stained kidney sections (**A**–**D**), representing score 0 (**A**); score 1 (**B**); score 2 (**C**); score 2 (**D**). Blue arrows: tubular epithelial ballooning. 200× magnification

In the kidneys, G3 (FBs alone) displayed mild to moderate lesions, characterized by tubular epithelial ballooning (Fig. 3C) and differed (*P* < 0.05) from G1 (Fig. 3A). However, FM did not significantly reduce (*P* > 0.05) the lesions induced by FBs (G7) (Fig. 3C). Furthermore, FM had no significant effect (*P* > 0.05) on the mild lesions (tubular epithelial ballooning) caused by AFs (G6) (Fig. 3B). In contrast, animals fed AFs + FBs and FM (G8) (Fig. 3A) showed absence of lesions in the evaluated animals (*P* < 0.05), compared to those that received only AFs + FBs (G4), which exhibited mild to moderate changes (Fig. 3D). Previous studies support renal involvement in FB toxicosis. Tubular epithelial vacuolization has been reported in piglets exposed to FB-contaminated diets (Grenier et al. 2011; Terciolo et al. 2019). In the present study, FB exposure was also associated with tubular epithelial degeneration. However, interstitial lymphocytic infiltration, which was described in some previous studies (Grenier et al. 2011; Terciolo et al. 2019), was not observed here. Aoyanagi et al. (2023) reported that a commercial adsorbent reduced glomerular atrophy in pigs fed an AF + FB + ZEN-contaminated diet. The renal effect of FM in the present study was observed only during combined exposure. This may reflect treatment-specific variation, toxin interaction, or limited statistical power.

**Fig. 4:**
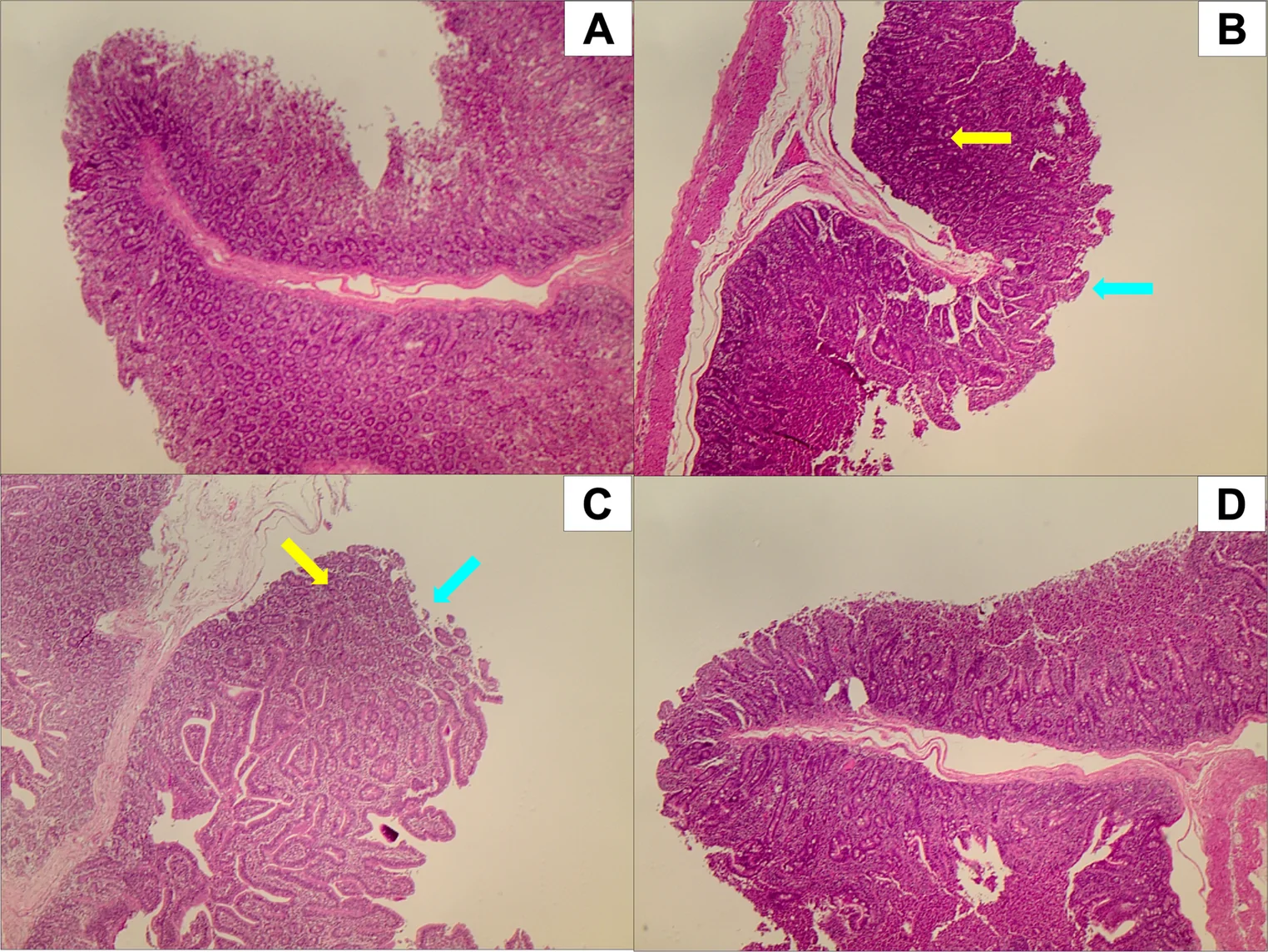
Representative microscopy photographs of hematoxylin- and eosin-stained jejunum sections (**A**–**D**), representing score 0 (**A**); score 2 (**B**); score 1 (**C**); score 1 (**D**). Yellow arrows: infiltrate inflammatory; Blue arrows: villous atrophy. 200× magnification

Animals fed AFs (G2) (Fig. 4B) or FBs (G3) (Fig. 4C) exhibited jejunal lesions ranging from absent to moderate, characterized by inflammatory infiltrate and villous atrophy. Although these findings were not significantly different (*P* > 0.05) from the control group (G1) (Fig. 4A), there was a tendency toward increased lesion severity in animals exposed to AFs or FBs. Moreover, jejunal lesions in animals fed AFs + FBs (G4) did not differ (*P* > 0.05) from the control group. The FM induced lower lesion scores (*P* < 0.05) in animals fed FBs (G7) (Fig. 4A), but had no significant effect (*P* > 0.05) on those fed AFs (G6) (Fig. 4D). Holanda et al. (2020) reported reduced jejunal crypt depth in pigs fed an AF + DON-contaminated diet. In that study, a yeast cell wall-based blend did not restore normal jejunal morphology. Terciolo et al. (2019) observed villous atrophy along with enterocyte flattening and apical necrosis in FB-exposed piglets. Fochesato et al. (2024) reported that broilers fed a diet contaminated with 0.506 mg/kg of AFB_1_ exhibited intestinal submucosal thickening. In the same study, animals receiving lyophilized *S. cerevisiae* RC016 (1 × 10^7^ cells/g) and *L. rhamnosus* RC007 (1 × 10^8^ cells/g) showed no intestinal changes. These findings support the intestine as a possible target of mycotoxin injury, but the jejunal response in the present study was variable.

Taken together, the histopathological findings indicate organ-dependent effects of AFs and FBs. AFs exposure was mainly associated with hepatic lesions, while FBs exposure caused predominantly pulmonary and renal lesions. FM reduced pulmonary lesion severity in FBs-exposed piglets and reduced kidney lesion severity under combined AFs + FBs exposure. Its effects on liver and jejunum were limited. These findings should be interpreted cautiously because the relatively small sample size per group may have limited the statistical power to detect significant differences, particularly in cases with subtle or variable responses among treatments.

### Performance of the analytical method for mycotoxins in plasma

The analytical performance parameters for plasma mycotoxin biomarkers are presented in Table 4. The LOD and LOQ in plasma for each mycotoxin were, respectively: AFM_1_ (0.04 and 0.15 ng/mL), AFP_1_ (0.57 and 1.88 ng/mL), AFQ_1_ (0.16 and 0.53), FB_1_ (0.05 and 0.16 ng/mL), and FB_2_ (0.06 and 0.2 ng/mL). The coefficient of determination (*r*^2^) for each mycotoxin calibration curve was greater than 0.99. The plasma samples were spiked with AFP_1_, AFM_1_, and AFQ_1_ in levels of 0.16 to 45.0 ng/mL, and FB_1_ and FB_2_ (0.39 to 225 ng/mL). The *R*_A_, SSE, and *R*_E_ for each mycotoxin are presented in Table 4. The *R*_A_ is the ratio of the calibration curve in the matrix (submitted through the sample preparation process) divided by the calibration curve in the solvent. The SSE expresses the matrix effect, calculated as the value of the calibration curve obtained using a blank extract (prepared using the sample preparation process) spiked with a standard, divided by the calibration curve in the solvent. The *R*_E_ is the yield obtained using *R*_A_ divided by the SSE. The *R*_A_ is commonly used as the total recovery of a method (Sulyok et al. 2006; Di Gregorio et al. 2017a). The apparent recoveries ranged from 71 to 89%. SSE values ranged from 65 to 116%. Extraction recoveries ranged from 61 to 116%. These values indicate that matrix effects were present, with signal suppression for FB_1_ and FB_2_ and signal enhancement for AF biomarkers. Therefore, matrix-matched calibration was used to correct quantitative results, as recommended for mycotoxin biomarker analysis (Di Gregorio et al. (2017a).

**Table 4:** Performance parameters of the analytical method for the determination of aflatoxins and fumonisins in pig’s plasma ^1^

Mycotoxin	RT (min)	Concentration range (ng/mL)	*R*_A_ (%)	SSE (%)	*R*_E_ (%)	LOD (ng/mL)	LOQ (ng/mL)
AFM_1_	4.31	0.16-10.0	73	115	63	0.04	0.15
AFP_1_	4.18	0.16-10.0	71	116	61	0.57	1.88
AFQ_1_	4.29	0.16-10.0	81	115	70	0.16	0.53
FB_1_	5.46	0.39-10.0	75	65	116	0.05	0.16
FB_2_	5.79	0.39-10.0	89	77	116	0.06	0.20

^1^ *N* = 3 per type of sample, in each spiked level

*RT* Retention time, *R*_A_: apparent recovery, *SSE* signal suppression/enhancement, *R*_E_ extraction recovery, *LOD* Limit of detection, *LOQ* Limit of quantification, *AFM*_1_ aflatoxin M_1,_*AFP*_1_ aflatoxin P_1,_*AFQ*_1_ aflatoxin Q_1_, *FB*_1_ fumonisin B_1_, *FB*_2_ fumonisin B_2_

### Mycotoxin residues in plasma and urine

Plasma biomarker results pooled across days 0, 14, and 28 are presented in Table 5. Animals fed uncontaminated diets, with or without FM (G1 and G5), had no quantifiable levels of the evaluated biomarkers. AFP_1_ and AFM_1_ were detected only in animals receiving AFs-contaminated diets (G2, G4, G6, and G8), while AFQ_1_ remained below the LOQ in all groups. These findings confirm systemic exposure to AFs in treated groups and indicate that AFP_1_ and AFM_1_ were the measurable AFs biomarkers under the conditions of this study. AFM_1_ concentrations did not differ between AFs-contaminated groups without FM (G2 and G4) and their respective FM-supplemented groups (G6 and G8) (*P* > 0.05). In contrast, AFP_1_ levels were below the LOQ in animals fed AF-contaminated diets and FM (G6 and G8), differing significantly (*P* < 0.05) from those fed AFs-contaminated diets without FM (G2 and G4). This result suggests that FM reduced plasma AFP_1_ in AFs-exposed piglets. However, the effect was not consistent across all AFs biomarkers because AFM_1_ was not reduced. FB_1_ and FB_2_ were detected mainly in FBs-exposed groups. Animals fed FBs-contaminated diets plus FM (G7 and G8) did not differ (*P* > 0.05) in plasma FB_1_ and FB_2_ levels compared to those not receiving FM (G3 and G4). Therefore, the biomarker data do not support a consistent reduction in systemic FBs exposure by FM. The detection of FB_2_ in G6 (AF and FM) may reflect the background FBs contamination measured in the basal diet (Supplementary Table S1), which should be acknowledged as a limitation for interpreting low plasma FB_2_ values in this group.

**Table 5:** Efficacy of a functional fermented milk (FM) in mitigating the toxic effects of individual and combined dietary exposure to aflatoxins (AFs) and fumonisins (FBs) on plasma mycotoxin biomarkers in piglets after 28 days of intoxication ^1^

Treatment group	AFs^2^ (mg/kg)	FBs^3^ (mg/kg)	FM^4^ (g/day)	AFP_1_ (ng/mL) Median (Range)	AFM_1_ (ng/mL) Median (Range)	AFQ_1_ (ng/mL) Median (Range)	FB_1_ (ng/mL) Median (Range)	FB_2_ (ng/mL) Median (Range)
G1 (Basal diet)	< 0.01 ^5^	0.96	0	< LOQ	< LOQ	< LOQ	< LOQ	< LOQ
G2	1.13	0.96	0	2.01 (0.94–3.95) ^a^	0.40 (0.07–0.66) ^a^	< LOQ	< LOQ	< LOQ
G3	< 0.01 ^5^	9.49	0	< LOQ	< LOQ	< LOQ	0.20 (0.08–0.27) ^ab^	0.25 (0.10–0.29) ^a^
G4	1.11	9.53	0	1.98 (0.94–2.03) ^a^	0.28 (0.07–0.47) ^a^	< LOQ	0.22 (0.08–0.92) ^a^	0.20 (0.10–0.25) ^a^
G5	< 0.01 ^5^	0.99	39 ^6^	< LOQ	< LOQ	< LOQ	< LOQ	< LOQ
G6	1.28	0.78	39 ^6^	< LOQ	0.39 (0.07–0.54) ^a^	< LOQ	< LOQ	0.10 (0.10–0.27) ^ab^
G7	< 0.01 ^5^	9.22	39 ^6^	< LOQ	< LOQ	< LOQ	0.08 (0.08–0.08) ^b^	0.10 (0.10–0.28) ^a^
G8	1.21	9.37	39 ^6^	< LOQ	0.39 (0.07–0.45) ^a^	< LOQ	0.08 (0.08–0.31) ^ab^	0.10 (0.10–0.22) ^ab^
*P*-value				0.002	0.001	-	0.009	0.109

^a−c^ Values within each column with no common superscript differ significantly by the Kruskal-Wallis test (*P* < 0.05)

^1^ Data are presented as medians and ranges (Q1–Q3) of 3 piglets per treatment, considering the pooled results obtained at 0, 14, and 28 days of intoxication

^2^ Sum of aflatoxins B_1_, B_2_, G_1_ and G_2_

^3^ Sum of fumonisins B_1_ and B_2_

^4^ Fermented milk containing 1.0% (*ww*) of cell biomasses of *Lacticaseibacillus rhamnosus* (1.0 × 10^10^ cells/g) and *Saccharomyces cerevisiae* (1.0 × 10^10^ cells/g) inactivated by autoclaving at 121 °C for 10 min

^5^ Limit of quantification for each aflatoxin

^6^ Equivalent to approximately 5% of the daily feed intake

*AFP*_1_ aflatoxin P_1_, *AFM*_1_ aflatoxin M_1_, AFQ_1_: aflatoxin Q_1_, *FB*_1_ fumonisin B_1_, *FB*_2_ fumonisin B_2_, *LOQ* limit of quantification (see Table 4 for LOQ values of each mycotoxin in plasma samples)

The reduction in AFP_1_ suggests partial effectiveness of FM in reducing AF bioavailability. This effect may be related to adsorption of AFs by heat-inactivated LR and SC in the gastrointestinal tract. However, the absence of a parallel reduction in AFM_1_, FB_1_, and FB_2_ indicates that the effect was biomarker-specific and incomplete. Vieira et al. (2024) reported reduced plasma levels of AFM_1_ and FBs in dairy cows treated with anti-mycotoxin adsorbents. Di Gregorio et al. (2017a) also demonstrated reduced AFB_1_-lysine adduct levels in pigs supplemented with an adsorbent. Compared with those studies, FM showed a narrower effect in the present experiment.

Analytical results of urinary biomarkers are presented in Table 6. The LOD and LOQ in urine for each mycotoxin were, respectively: AFM_1_ (0.01 and 0.03 ng/mL), AFP_1_ (0.19 and 0.65 ng/mL), AFQ_1_ (0.22 and 0.72), FB_1_ (0.08 and 0.27 ng/mL), and FB_2_ (0.13 and 0.42 ng/mL). Results were pooled across sampling days 0, 7, 14, 21, and 28, and expressed as ng/mg creatinine. AFM_1_ and AFQ_1_ were detected only in animals fed AFs-contaminated diets, whereas AFP_1_ was not detected in any group. FB_1_ and FB_2_ were predominantly detected in animals fed FBs-contaminated diets. However, animals receiving AFs-contaminated diets alone (G2) also showed low median concentrations of FB_1_ and FB_2_ (0.14 and 0.22 ng/mg creatinine, respectively), while those fed AFs-contaminated feed plus FM (G6) also excreted FB_2_ at median of 0.30 ng/mg creatinine. As observed for plasma, these low levels of FB_1_ and FB_2_ in the urine of piglets from G2 and G6 are likely due to FBs contamination in the basal diet (Supplementary Table S1), thus limiting the interpretation of low urinary FBs values in those groups. Overall, these findings demonstrate that AFM_1_, AFQ_1_, FB_1_, and FB_2_ can be detected in urine and may serve as useful biomarkers of exposure to AFs and FBs in pigs. AFM_1_ and AFQ_1_ concentrations did not differ (*P* > 0.05) between animals fed AFs-contaminated diets without FM (G2 and G4) and their respective FM-treated groups (G6 and G8). These findings suggest that FM did not effectively reduce urinary AFs biomarkers under the experimental conditions evaluated. Median urinary FB_2_ levels were 0.69 ng/mL creatinine in animals fed AFs + FBs-contaminated diets (G4), differing significantly (*P* < 0.05) from animals receiving the same diet with FM (G8), which showed median levels of 0.46 ng/mg creatinine. In contrast, urinary FB_1_ levels did not differ (*P* > 0.05) between animals fed FBs-contaminated diets with FM (G7 and G8) and those not receiving FM (G3 and G4). These findings suggest that FM had limited effects on urinary FBs biomarkers, although it was associated with reduced urinary FB_2_ levels.

**Table 6:** Efficacy of a functional fermented milk (FM) in mitigating the toxic effects of individual and combined dietary exposure to aflatoxins (AFs) and fumonisins (FBs) on urinary mycotoxin biomarkers in piglets after 28 days of intoxication ^1^

Treatment group	AFs^2^ (mg/kg)	FBs^3^ (mg/kg)	FM^4^ (g/day)	AFP_1_ (ng/mg creatinine) Median (Range)	AFM_1_ (ng/mg creatinine) Median (Range)	AFQ_1_ (ng/mg creatinine) Median (Range)	FB_1_ (ng/mg creatinine) Median (Range)	FB_2_ (ng/mg creatinine) Median (Range)
G1 (Basal diet)	< 0.01 ^5^	0.96	0	< LOQ	< LOQ	< LOQ	< LOQ	< LOQ
G2	1.13	0.96	0	< LOQ	22.96 (5.11–38.60) ^a^	3.12 (1.38–4.98) ^a^	0.14 (0.12–0.18) ^b^	0.22 (0.19–0.28) ^c^
G3	< 0.01 ^5^	9.49	0	< LOQ	0.01 (0.01–0.01) ^b^	0.42 (0.36–0.51) ^b^	8.65 (2.50–25.74) ^a^	0.28 (0.24–0.32) ^bc^
G4	1.11	9.53	0	< LOQ	11.46 (3.96–38.06) ^a^	2.74 (0.72–7.18) ^a^	29.21 (19.760–55.70) ^a^	0.69 (0.42–1.16) ^a^
G5	< 0.01 ^5^	0.99	39 ^6^	< LOQ	< LOQ	< LOQ	< LOQ	< LOQ
G6	1.28	0.78	39 ^6^	< LOQ	18.56 (0.25–33.84) ^a^	3.53 (0.72–5.25) ^a^	< LOQ)	0.30 (0.23–0.32) ^bc^
G7	< 0.01 ^5^	9.22	39 ^6^	< LOQ	0.01 (0.01–0.01) ^b^	0.45 (0.42–0.51) ^b^	15.71 (5.35–33.53) ^a^	0.35 (0.26–0.60) ^b^
G8	1.21	9.37	39 ^6^	< LOQ	20.62 (7.67–34.22) ^a^	3.87 (1.53–5.73) ^a^	20.16 (9.40–45.36) ^a^	0.46 (0.22–0.96) ^b^
*P*-value				-	< 0.001	< 0.001	< 0.001	< 0.001

^a−c^ Values within each column with no common superscript differ significantly by the Kruskal-Wallis test (*P* < 0.05)

^1^ Data are presented as medians and ranges (Q1–Q3) of 3 piglets per treatment, considering the pooled results obtained at 0, 7, 14, 21, and 28 days of intoxication

^2^ Sum of aflatoxins B_1_, B_2_, G_1_ and G_2_

^3^ Sum of fumonisins B_1_ and B_2_

^4^ Fermented milk containing 1.0% (*w*/*w*) of cell biomasses of *Lacticaseibacillus rhamnosus* (1.0 × 10^10^ cells/g) and *Saccharomyces cerevisiae* (1.0 × 10^10^ cells/g) inactivated by autoclaving at 121 °C for 10 min

^5^ Limit of quantification for each aflatoxin

^6^ Equivalent to approximately 5% of the daily feed intake

*AFP*_1_aflatoxin P_1_ , *AFM*_1_aflatoxin M_1_ , *AFQ*_1_ aflatoxin Q_1_ , *FB*_1_ fumonisin B_1_, *FB*_2_ fumonisin B_2_, *LOQ* limit of quantification (0.03, 0.65, 0.72, 0.27 and 0.42 ng/mL for AFM_1_, AFP_1_, AFQ_1_, FB_1_ and FB_2_, respectively)

In the review by Tkaczyk and Jedziniak (2021), the authors did not find any evidence of detectable levels of AFP_1_ in urine of pigs, which corroborate the findings in the present study and confirm that AFP_1_ is not a suitable urinary biomarker of AFs exposure. Conversely, Vieira et al. (2024) reported reductions in urinary AFM_1_ and FBs biomarkers in dairy cows fed diets contaminated with multiple mycotoxins and supplemented with commercial adsorbents. Although previous studies have demonstrated the efficacy of other adsorbents in reducing urinary AFs and FBs biomarkers, FM did not show similar effects in piglets under the experimental conditions evaluated. The combined results show that AFs and FBs produced different toxicological patterns in piglets. AFs exposure reduced body-weight gain and feed consumption and was associated with hepatic biochemical and histological alterations. FBs exposure produced more evident microscopic lesions in the lung and kidney. Combined AFs + FBs exposure caused alterations in ALT and creatinine. FM showed partial and organ-specific effects. It did not consistently improve growth performance or serum biochemistry. It reduced lung lesion severity in FBs-exposed piglets and kidney lesion severity under combined AFs + FBs exposure. It also reduced plasma AFP_1_, and urinary FB_2_ in in piglets exposed to AFs and AF + FBs, respectively, although FM did not consistently reduce AFM_1_, FB_1_, or FB_2_ in plasma and AFM_1_, AFQ_1_, or FB_1_ in urine. These findings suggest partial reduction of AFs and FBs bioavailability and limited protection against selected organ lesions. However, several limitations of the present study should be acknowledged. Firstly, a limited number of animals per group was used in the experimental design, given the large amounts of AFs- and FBs-contaminated culture materials required for feed preparation. Secondly, the inability to determine the exact mycotoxin intake due to feed wastage by the animals may also have impacted the effective dose of each experimental animal. Thirdly, the experiment was not designed to assess the effect of different FM or microbial biomass inclusion levels on the toxicity and biomarker parameters associated with AFs and FBs exposure. Finally, the FM was administered approximately 5 min before feed delivery, to ensure that the microbial biomass reached the gastrointestinal tract (GIT) before the ingestion of the contaminated feed, thereby increasing the likelihood of contact between microbial binding sites and dietary mycotoxins during passage through GIT. Although this procedure is similar to other *in vivo* studies reported in the literature for efficacy evaluation of mineral or organic products, in which the adsorbent is mixed with the feed administered to the animals, it was not possible to evaluate if the 5 min timing before feeding and the daily amount of FM were sufficient to create meaningful GIT contact between mycotoxins and microbial binding sites, neither directly measure the mycotoxin fecal excretion of AFs and FBs. These factors may have reduced the statistical power of the study and increased the influence of individual variability.

## Conclusions

In conclusion, AFs and FBs induced distinct toxicological effects in piglets, with AFs primarily affecting growth performance and hepatic parameters, while FBs were associated with pulmonary and renal lesions. Combined exposure intensified some toxicological responses, particularly hepatic alterations. The FM containing heat-inactivated *L. rhamnosus* and *S. cerevisiae* showed partial and organ-specific protective effects, reducing pulmonary and renal lesions, as well as plasma AFP_1_ and urinary FB_2_ levels. However, FM did not consistently improve animal performance or biochemical parameters. These findings suggest a limited but observable capacity of FM to modulate mycotoxin toxicity in pigs. Further studies with larger group sizes, different microorganisms and mycotoxin dosage, dose-response designs, and direct adsorption/bioavailability measurements are needed to confirm the protective potential of FM containing heat-inactivated microorganisms’ biomasses.

## Supplementary Information

Below is the link to the electronic supplementary material.

Supplementary Material 1

## Data Availability

No datasets were generated or analysed during the current study.
