# Supplementary material for: Efficacy evaluation of a fermented milk containing inactivated biomasses of Lacticaseibacillus rhamnosus and Saccharomyces cerevisiae to ameliorate the toxic effects of dietary aflatoxins and fumonisins in pigs: a pilot study
Source: Mycotoxin Res. 2026 Sep 4;42(4):65. doi: 10.1007/s12550-026-00677-7 (PMC13545053; doi:10.1007/s12550-026-00677-7)
Supplement: Supplementary file 1 [file 12550_2026_677_MOESM1_ESM.docx]

**Supplementary material:**

**Table S1**

Dietary treatments groups and respective concentrations of aflatoxins and fumonisins in the experimental feed^1^.

| Treatment group^2^ | AFB_1_ (mg/kg) | AFB_2_ (mg/kg) | AFG_1_ (mg/kg) | AFG_2_ (mg/kg) | TAFs (mg/kg) | FB_1_ (mg/kg) | FB_2_ (mg/kg) | TFBs (mg/kg) |
| --- | --- | --- | --- | --- | --- | --- | --- | --- |
| G1 | <LOQ | <LOQ | <LOQ | <LOQ | <LOQ | 0.52 | 0.44 | 0.96 |
| G2 | 0.64 | 0.07 | 0.39 | 0.03 | 1.13 | 0.54 | 0.42 | 0.96 |
| G3 | <LOQ | <LOQ | <LOQ | <LOQ | <LOQ | 7.60 | 1.89 | 9.49 |
| G4 | 0.70 | 0.03 | 0.35 | 0.03 | 1.11 | 7,48 | 2.05 | 9.53 |
| G5 | <LOQ | <LOQ | <LOQ | <LOQ | <LOQ | 0.74 | 0.25 | 0.99 |
| G6 | 0.76 | 0.04 | 0.45 | 0.02 | 1.27 | 0.46 | 0.32 | 0.78 |
| G7 | <LOQ | <LOQ | <LOQ | <LOQ | <LOQ | 7.14 | 2.08 | 9.22 |
| G8 | 0.75 | 0.05 | 0.39 | 0.02 | 1.21 | 6.79 | 2.58 | 9.37 |

^1^ Mean of duplicate samples analyzed individually.

^2^ G1: Control, only basal diet (BD); G2: BD + aflatoxins (AFs); G3: BD + fumonisins (FBs); G4: BD + AFs + FBs; G5: BD + Fermented milk containing 1.0% (w/w) of cell biomasses of *Lacticaseibacillus rhamnosus* (1.0 x 10^10^ cells/g) and *Saccharomyces cerevisiae* (1.0 x 10^10^ cells/g) inactivated by autoclaving at 121 °C for 10 min (FM); G6: BD + AFs + FM; G7: BD + FBs + FM; G8: BD + AFs + FBs + FM.

AFB_1_: aflatoxin B_1_; AFB_2_: aflatoxin B_2_; AFG_1_: aflatoxin G_1_; AFG_2_: aflatoxin G_2_; TAFs: total aflatoxins (sum of AFB_1_, AFB_2_, AFG_1_ and AFG_2_); FB_1_: fumonisin B_1_; FB_2_: fumonisin B_2_; TFBs: total fumonisins (sum of FB_1_ and FB_2_); LOQ: Limit of quantification (0.01 mg/kg for each aflatoxin or fumonisin).

**Table S2**

Chromatographic conditions used in the analyses for determination of aflatoxins and fumonisins in pig’s plasma and urine.

| Parameter | Description |
| --- | --- |
| Column temperature | 40^o^ C |
| Sample temperature | 15^o^ C |
| Mobile phase | Water (eluent A) and acetonitrile (eluent B), both containing 5.0 mM of ammonium acetate + 0.1% of acetic acid |
| Gradient elution | For elution of injected samples, percentage of eluent A was kept at 98% for 1.0 min. After this period, percentage of eluent B was linearly raised to 30% over 4.0 min (5.0 min). Then, eluent B was increased to 96% over 1.5 min, followed by a hold time of 0.5 min (7.0 min). After that, percentage of eluent B was reduced to 2% over 1.0 min (8.0 min), and the column re-equilibrated to the initial conditions for 2.0 min |
| Flow rate | 0.6 mL/min |
| Total chromatographic run time | 10.0 min |

**Table S3**

Mass spectrometry (MS) and analytical parameters used for the determination of aflatoxins and fumonisins in pig’s plasma and urine.

| Mycotoxin | Mass (g/mol) | Molecular ion | Transition (*m/z*) | Cone voltage (V) | Collision energy (V) |
| --- | --- | --- | --- | --- | --- |
| AFM_1_ | 328.3 | [M+H]^+^ | 328.9 > 273.1 ^a^ | 52 | 24 |
|  |  |  | 329.0 > 229.0 ^b^ |  | 38 |
| [^13^C_17_]-AFM_1_ | 329.1 | [M+H]^+^ | 346.1 > 288.1 | 52 | 25 |
| AFP_1_ | 298.3 | [M+H]^+^ | 299.3 > 271.0 ^a^ | 56 | 30 |
|  |  |  | 299.3 > 215.1 ^b^ |  | 22 |
| AFQ_1_ | 328.3 | [M+H]^+^ | 329.3 > 206.1 ^a^ | 60 | 34 |
|  |  |  | 329.3 > 177.2 ^b^ |  | 24 |
| FB_1_ | 721.8 | [M+H]^+^ | 722.5 > 334.0 ^a^ | 50 | 40 |
|  |  |  | 722.5 > 352.0 ^b^ |  | 30 |
| [^13^C_34_]-FB_1_ | 755.6 | [M+H]^+^ | 756.6 > 374.4 | 50 | 30 |
| FB_2_ | 705.8 | [M+H]^+^ | 706.5 > 336.2 ^a^ | 30 | 35 |
|  |  |  | 706.5 > 318.3 ^b^ |  | 40 |

AFM_1_: aflatoxin M_1_; AFP_1_: aflatoxin P_1_; AFQ_1_: aflatoxin Q_1_; FB_1_: fumonisin B_1_; FB_2_: fumonisin B_2_.

^a^ Transitions used for quantification; ^b^ Transitions used for confirmation.

**Table S4**

Significant main effects between the functional fermented milk (FM^1^) and the dietary exposure to aflatoxins (AFs^2^) and fumonisins (FBs^3^) on body weight gain and feed consumption of piglets over 28 days of intoxication.

| Parameter | Main effect | Response | |
| --- | --- | --- | --- |
| Body weight gain (kg)^4^ | AFs | Absent | 14.21 ± 2.12 ^a^ |
|  |  | Present | 10.59 ± 2.65 ^b^ |
| Feed consumption (kg)^4^ | AFs | Absent | 23.40 ± 2.28 ^a^ |
|  |  | Present | 18.75 ± 3.77 ^b^ |

^a-c^ In the same column, means followed by different lower case superscript letters differ significantly by the Tukey test (*P* < 0.05).

^1^ Fermented milk containing 1.0% (*w*/*w*) of cell biomasses of *Lacticaseibacillus rhamnosus* (1.0 x 10^10^ cells/g) and *Saccharomyces cerevisiae* (1.0 x 10^10^ cells/g) inactivated by autoclaving at 121 °C for 10 min.

^2^ Sum of aflatoxins B_1_, B_2_, G_1_ and G_2_.

^3^ Sum of fumonisins B_1_ and B_2_.

^4^ Data are means ± SD (*N* = 18) of 3 piglets per treatment evaluated at 0, 14 and 28 days of intoxication.

**Table S5**

Significant main effects and interactions between the functional fermented milk (FM^1^) and the dietary exposure to aflatoxins (AFs^2^) and fumonisins (FBs^3^) on serum alanine aminotransferase (ALT) and creatinine of piglets over 28 days of intoxication.

| Parameter | Interaction / Main effect | Response | | |
| --- | --- | --- | --- | --- |
| ALT (U/L)^4^ | AFs x FBs |  | AFs (absent) | AFs (present) |
|  |  | FBs (absent) | 98.61 ± 32.91 ^Aa^ | 90.72 ± 22.33 ^Ab^ |
|  |  | FBs (present) | 92.61 ± 24.98 ^Ba^ | 127.2 ± 31.52 ^Aa^ |
|  | AFs x FM |  | AFs (absent) | AFs (present) |
|  |  | FM (absent) | 81.94 ± 22.49 ^Bb^ | 109.22 ± 34.26 ^Aa^ |
|  |  | FM (present) | 109.3 ± 28.71 ^Aa^ | 108.67 ± 31.96 ^Aa^ |
| Creatinine (mg/dL)^4^ | AFs x FBs |  | AFs (absent) | AFs (present) |
|  |  | FBs (absent) | 0.647 ± 0.070 ^Aa^ | 0.684 ± 0.075 ^Aa^ |
|  |  | FBs (present) | 0.653 ± 0.071 ^Aa^ | 0.595 ± 0.054 ^Bb^ |
|  | FBs x FM |  | FM (absent) | FM (present) |
|  |  | FBs (absent) | 0.687 ± 0.075 ^Aa^ | 0.644 ± 0.068 Aa |
|  |  | FBs (present) | 0.604 ± 0.070 ^Ab^ | 0.643 ± 0.063 ^Aa^ |
| ALP (U/L)^4^ | AFs | Absent | 663.6 ± 252.1 ^b^ |  |
|  |  | Present | 842.1 ± 308.1 ^a^ |  |
| ALB (g/dL)^4^ | FM | Absent | 680.9 ± 242.5 ^b^ |  |
|  |  | Present | 824.8 ± 324.7 ^a^ |  |
|  | FBs | Absent | 3.042 ± 0.305 ^a^ |  |
|  |  | Present | 2.847 ± 0.292 ^b^ |  |

^A-C^ In the same row, means followed by different capital superscript letters differ significantly by the Tukey test (*P* < 0.05).

^a-c^ In the same column, means followed by different lower case superscript letters differ significantly by the Tukey test (*P* < 0.05).

^1^ Fermented milk containing 1.0% (*w*/*w*) of cell biomasses of *Lacticaseibacillus rhamnosus* (1.0 x 10^10^ cells/g) and *Saccharomyces cerevisiae* (1.0 x 10^10^ cells/g) inactivated by autoclaving at 121 °C for 10 min.

^2^ Sum of aflatoxins B_1_, B_2_, G_1_ and G_2_.

^3^ Sum of fumonisins B_1_ and B_2_.

^4^ Data are means ± SD (*N* = 18) of 3 piglets per treatment evaluated at 0, 14 and 28 days of intoxication.
